# The influence of work-life balance on teacher performance in Zhengzhou, China

**DOI:** 10.3389/fpsyg.2025.1586128

**Published:** 2025-09-10

**Authors:** Meng Li, Ahmad Albattat

**Affiliations:** ^1^Academy of Marxism, Henan Open University, Zhengzhou, China; ^2^School of Global Hospitality and Tourism, Asia Pacific University of Technology & Innovation, Kuala Lumpur, Malaysia

**Keywords:** conservation of resources theory, work-life balance, stress levels, emotional intelligence, teacher performance, Zhengzhou, smart-PLS

## Abstract

**Introduction:**

This study examines the complex interconnections between work-life balance elements, stress levels, emotional intelligence, and teacher effectiveness, contextualised within the Conservation of Resources (COR) paradigm. The research examines the impact of resource conservation and emotional regulation on performance, emphasising stress levels as a mediator and emotional intelligence as a moderator.

**Methods:**

A quantitative methodology was utilised, involving data collection from 300 educators in Zhengzhou, China, and analysis by Structural Equation Modelling using Smart-PLS. The findings indicated substantial direct and indirect correlations.

**Results:**

Social support availability (SSA) exhibited a substantial direct effect on stress levels (T = 4.648, *P* = 0.000), whereas work environment harmony (WM) and SSA notably affected teacher performance (T = 2.099, *P* = 0.036; T = 2.626, *P* = 0.009). Mediation analysis indicated complete mediation for AFP âƒ™ SL âƒ™ TP (H3, T = 3.021, *P* = 0.003) and complementing partial mediation for SSA âƒ™ SL âƒ™ TP (H4, T = 2.574, *P* = 0.010). Moderation analysis revealed that emotional intelligence substantially mitigated the effect of stress on teacher performance (H11, T = 4.129, *P* = 0.000).

**Discussion:**

These findings expand COR theory by associating resource conservation with emotional intelligence, providing theoretical insights and practical recommendations for developing supportive settings that improve teacher performance. Educational officials in Zhengzhou are urged to create interventions aimed at reducing stress and enhancing emotional intelligence to attain sustained improvements in teacher performance.

## 1 Introduction

### 1.1 The motivation for the study

Work-life balance (WLB) has become a pivotal subject in international academic and policy discourse because of its substantial impact on employee well-being and organisational performance. In the educational sector, especially in Zhengzhou, China, sustaining a healthy work-life balance for educators is crucial for delivering high-quality education and promoting student achievement. Educators are crucial in moulding the future workforce, and their effectiveness directly influences student achievements and the overall advancement of human capital, in accordance with China’s educational reform objectives, such as the “Double Reduction” strategy. Nonetheless, the escalating demands placed on educators in Zhengzhou, including onerous workloads, stress, and insufficient institutional support, have posed hurdles to attaining work-life balance (Hillman and Ren, 2024). These problems substantially impact teachers’ performance, motivation, and overall well-being. This study is motivated by the pressing necessity to tackle these concerns due to their impact on the sustainability and efficacy of Zhengzhou’s education system.

### 1.2 General issues related to work-life balance in Zhengzhou, China

The difficulties of achieving work-life balance for teachers in Zhengzhou are exacerbated by increasing professional responsibilities (Meng et al., 2024). Educators are anticipated to oversee a variety of obligations, encompassing curriculum development, extracurricular engagements, administrative tasks, and student guidance (Javed and Akhter, 2024). These obligations are frequently exacerbated by systemic issues, including substantial class sizes, insufficient teaching resources, and inadequate assistance from school administrators. Reports indicate that educators in Zhengzhou, especially in metropolitan areas, experience some of the greatest levels of occupational stress, frequently ascribed to the competitive academic milieu and societal demands (Li et al., 2024). The issue in rural areas is worsened by inadequate family-friendly regulations and rigid work arrangements, resulting in discrepancies in work-life balancing experiences between urban and rural instructors (Nicer and Faller, 2024). These concerns result in negative consequences, including diminished teacher effectiveness, reduced work satisfaction, and possible decreases in the quality of education provided (Wang et al., 2024).

### 1.3 Problems in the education sector related to the research topic

A significant concern in Zhengzhou’s education sector is the detrimental effect of work-life imbalance on teacher effectiveness (Meng et al., 2024). Educators frequently contend with the conflicting demands of their professional and personal lives, resulting in increased stress and reduced efficacy in the classroom (Conte et al., 2024). Rigid work schedules and inadequate workload management prevent many instructors from dedicating adequate time to personal duties, exacerbating their stress and discontent. The inadequate implementation of family-friendly policies in rural schools intensifies systemic imbalances, obstructing teachers in these environments from attaining a good work-life balance (Zhang et al., 2024). Emotional intelligence, a potential mitigating factor for these issues, is underutilised and inadequately examined in Zhengzhou’s schools, resulting in educators being ill-prepared to manage stress efficiently (Rakhmetov et al., 2024). Confronting these obstacles is essential for enhancing teacher effectiveness, retaining skilled educators, and fulfilling China’s educational reform objectives.

### 1.4 Research gap and objectives

Although work-life balance is acknowledged as a vital element in the teaching profession, considerable gaps remain in comprehending its intricate impacts on teacher performance within the Zhengzhou setting (Zhu, 2024). Prior research has either generalised results across other professions or neglected critical characteristics, such emotional intelligence and stress, as moderating and mediating factors, respectively. Moreover, scant research has investigated geographical inequalities between urban and rural schools or differences in institutional kinds, such as public and private schools. This study aims to address these gaps by examining the impact of work-life balance factors—such as flexible work hours, workload management, availability of family-friendly policies, and support from school administration—on teacher performance. Stress levels are analysed as a mediator, while emotional intelligence is evaluated as a moderator. The study examines a varied cohort of educators from different educational tiers and institutional environments in Zhengzhou to guarantee practical and contextually pertinent results.

### 1.5 Novelty and contribution of the study

This study presents a unique viewpoint by utilising the Conservation of Resources (COR) Theory to examine the relationship among work-life balance, stress, and teacher performance. By including emotional intelligence as a moderating variable, the research offers a thorough understanding of the impact of internal and external resources on teacher outcomes, a domain inadequately examined in previous studies. The study utilises sophisticated analytical methods, providing substantial insights into the interactions of various variables. The findings are anticipated to assist policymakers and school administrators in formulating programmes that promote work-life balance, alleviate teacher stress, and boost overall educational outcomes. These tactics are essential for tackling the distinct issues encountered by educators in Zhengzhou, particularly in rural and public school environments.

The subsequent sections of this work are organised as follows: Section 2 presents a comprehensive literature review, outlining the theoretical framework of the investigation. Section 3 delineates the research methodology, encompassing sample tactics and data collection techniques. Section 4 delineates the findings, while Section 5 examines their implications for policy and practice. The publication finishes with suggestions for further research and recognition of the study’s shortcomings.

## 2 Literature review

### 2.1 Conservation of resources (COR) theory as an underpinning framework

This research utilises the Conservation of Resources (COR) Theory, established by Hobfoll (1989), as its principal theoretical foundation. COR Theory asserts that humans endeavour to get, save, and safeguard resources like time, energy, and emotional health. Stress occurs when these resources are jeopardised, depleted, or inadequate to fulfil demands. In the educational sector, where educators encounter complex and resource-demanding challenges, COR Theory offers an optimal framework to examine the impact of resource depletion on teacher effectiveness.

In Zhengzhou, China, governmental policies emphasise enhancing work-life balance and teacher welfare as components of comprehensive educational reforms under the “Double Reduction” initiative, which seeks to alleviate teacher workloads and student stress (Lian, 2024). Educators in Zhengzhou must manage both instructional responsibilities and administrative duties while balancing personal commitments, frequently under considerable resource limitations. The COR theory corresponds with this dynamic, clarifying how resource depletion due to excessive work demands results in stress and offering solutions for resource conservation to improve teacher effectiveness (Gu et al., 2024).

Educators in Zhengzhou often face resource-related pressures, including as inflexible work hours, excessive workloads, inadequate family-friendly policies, and insufficient institutional support (Gaborova, 2024). These issues are particularly evident in public schools, where the execution of policies and administrative support may fluctuate. Consequently, COR Theory serves as the foundation for this study’s examination of the interconnections between work-life balance elements, stress, emotional intelligence, and teacher effectiveness.

### 2.2 Teacher performance as the dependent variable

The efficacy of teachers is a pivotal factor influencing student success and the overall quality of education. Effective teacher performance encompasses delivering captivating classes, promoting student engagement, and attaining educational goals. Nonetheless, stress and resource depletion can compromise teachers’ ability to perform effectively (Hobfoll, 2021). Studies demonstrate that stress negatively impacts teacher performance by reducing their concentration, vitality, and emotional ability to handle classroom interactions (Ntim et al., 2023).

Empirical evidence indicates that enhancing work-life balance elements can substantially improve teacher performance. Flexible work hours, task management, and helpful school administration are elements that mitigate stress, enabling teachers to focus their energies on professional obligations (Chan et al., 2021). This study asserts that teacher performance is directly affected by work-life balance elements and indirectly influenced by stress levels, with emotional intelligence acting as a moderator.

### 2.3 Emotional intelligence as a moderator

Emotional intelligence (EI) moderates the correlation between stress levels and teacher effectiveness by improving resource conservation and management (D’Souza et al., 2022). Educators possessing elevated emotional intelligence are more adept at regulating emotions, managing stress, and sustaining resilience in difficult situations. COR Theory posits that persons with elevated emotional intelligence possess enhanced capabilities to conserve and allocate resources efficiently, hence alleviating the adverse impacts of stress on performance (Peethambaran and Naim, 2024).

Empirical evidence substantiates the moderating function of emotional intelligence (EI). Estrada et al. (2021) discovered that emotional intelligence mitigates the negative effects of stress on teacher performance, allowing educators to sustain concentration and interact effectively with pupils despite workplace difficulties. Likewise, Blendea et al. (2024) shown that educators with elevated emotional intelligence display more adaptability and resilience, hence improving their capacity to manage stress-inducing circumstances. This study proposes that emotional intelligence enhances the favourable impacts of work-life balance elements on teacher performance by mitigating stress impact.

### 2.4 Stress levels as a mediator

This study conceptualises stress as a mediating variable that connects work-life balance parameters to teacher performance. COR Theory posits that stress occurs when the demands for resources surpass their availability, resulting in resource depletion and a decline in well-being. Stress diminishes cognitive and emotional functioning, thus impacting teachers’ effectiveness (Bardach et al., 2022).

Flexible work hours, task management, family-friendly policies, and assistance from school administration are essential components for achieving work-life balance that alleviate stress. Flexible work options enable instructors to manage their time effectively, hence minimising role conflict and related stress (Ghali-Zinoubi et al., 2024). Efficient task management prevents instructors from becoming inundated with their duties, preserving mental and physical resources (Minihan et al., 2022). Family-friendly policies facilitate instructors in reconciling personal and professional obligations, alleviating stress arising from work–family conflicts (Jongruck and Vyas, 2024). Assistance from school administration offers institutional resources and emotional support, alleviating occupational stress (Wang, 2024).

Empirical research corroborates these associations. Zhao et al. (2022) illustrated that stress mediates the influence of workload management on teacher performance, whereas Alzoubi and Alzoubi (2023) emphasised the significance of administrative support in mitigating stress and enhancing performance. This research expands upon previous findings by investigating the mediating influence of stress on several work-life balance characteristics within the Zhengzhou setting.

Based on the theoretical framework and literature review, the following hypotheses are proposed:

### 2.5 Moderation hypotheses

1. H1: Emotional Intelligence (EI) moderates the relationship between Stress Levels (SL) and Teacher Performance (TP).

### 2.6 Indirect hypotheses (mediation)

1. H2: Stress Levels (SL) mediates the relationship between Flexible Work Hours (FWH) and Teacher Performance (TP).
2. H3: Stress Levels (SL) mediates the relationship between Workload Management (WM) and Teacher Performance (TP).
3. H4: Stress Levels (SL) mediates the relationship between Availability of Family-Friendly Policies (AFP) and Teacher Performance (TP).
4. H5: Stress Levels (SL) mediates the relationship between Support from School Administration (SSA) and Teacher Performance (TP).

## 3 Methodology

### 3.1 Research design

This research utilises a quantitative approach to investigate the correlations between work-life balance parameters, stress levels, emotional intelligence, and teacher performance. A quantitative methodology enables the gathering of numerical data to identify patterns, linkages, and causal links (Creswell et al., 2021). This approach corresponds with the study’s aim of evaluating theoretical hypotheses based on the Conservation of Resources (COR) theory. Data were gathered using a structured survey tool, ensuring uniformity and facilitating statistical analysis to confirm the proposed correlations.

### 3.2 Population and sampling

The study population comprises secondary school educators employed in both public and private institutions in Zhengzhou, China. This demographic was selected because of the escalating expectations placed on educators within China’s education system, especially in the swiftly urbanising Zhengzhou area, rendering it an appropriate backdrop for examining the influence of work-life balance elements on teacher performance.

A desired sample size of 400 respondents was established, conforming to the guidelines for structural equation modelling (SEM), which necessitates a minimum of 200 to 300 participants to guarantee reliable parameter values and model fit (Hair and Alamer, 2022). Stratified random sampling was utilised to guarantee representativeness, with strata determined by school type (public versus private) and geographic location (urban versus suburban), so ensuring sample variety.

Of the 400 questionnaires issued, 320 were returned, resulting in a response rate of 80%. This response rate is deemed high for survey-based research and adequate for SEM analysis (Byrne, 2022). The sample size was considered sufficient to fulfil the study’s aims and satisfy statistical criteria.

### 3.3 Data collection

Data were gathered through a standardised self-administered survey disseminated from May to July 2024. The poll aimed to assess respondents’ views on work-life balance elements, stress levels, emotional intelligence, and teacher performance. It was disseminated electronically through email and local school communication networks to augment participation.

The survey comprised a cover letter detailing the study’s objective, securing informed consent, and assuring confidentiality. Participants were allotted 4 weeks to complete the survey, with weekly follow-up reminders dispatched to enhance the response rate. The implementation of electronic surveys enhanced accessibility and diminished administrative burdens.

### 3.4 Measures and instruments

The survey questionnaire comprised validated scales modified from prior studies to assess the components. Each item was evaluated using a five-point Likert scale (1 = strongly disagree to 5 = strongly agree), ensuring uniformity between assessments. The scales’ reliability and validity were established through previous research and revalidated for this study. The survey instruments were administered in the Chinese language to ensure congruence with the linguistic context of the study population. In order to maintain both conceptual and linguistic precision, the original English scales were subjected to a meticulous forward-backward translation procedure. Initially, two bilingual experts conducted independent translations of the scales into Chinese, which was subsequently followed by a reconciliation phase aimed at addressing any discrepancies that arose. Subsequently, a third translator conducted a back-translation of the Chinese version into English, intentionally omitting any reference to the original scales. This approach enabled the research team to assess the semantic equivalence effectively.

The constructs in this study were measured using validated scales adapted from prior research, ensuring reliability and consistency. Flexible Work Hours (FWH) were assessed using a four-item scale from Mohiya (2024) with a Cronbach’s alpha of 0.88. Workload Management (WM) was measured with a five-item scale adapted from Giotopoulos et al. (2024) achieving a Cronbach’s alpha of 0.90. Availability of Family-Friendly Policies (AFP) was captured using a five-item scale developed by Chen (2023) with a Cronbach’s alpha of 0.89. Support from School Administration (SSA) was evaluated using a six-item scale from (Manjo, 2024) demonstrating a Cronbach’s alpha of 0.92. Stress Levels (SL) were measured using a six-item scale adapted from Kosherbayeva et al. (2024) with a Cronbach’s alpha of 0.91. Emotional Intelligence (EI) was assessed with a four-item scale adapted from Britwum et al. (2024), achieving a Cronbach’s alpha of 0.93. Lastly, Teacher Performance (TP) was measured using a seven-item scale from (Hobfoll, 2021) with a Cronbach’s alpha of 0.95, confirming the high reliability of all scales used in the study.

The reliability of each scale was evaluated using Cronbach’s alpha, with all results surpassing the recommended threshold of 0.70, signifying internal consistency (Hair and Alamer, 2022). Confirmatory factor analysis (CFA) was performed to verify validity, exhibiting satisfactory factor loadings and model fit.

### 3.5 Data analysis

Data analysis was conducted utilising SPSS version 28 for initial assessments, encompassing data cleansing, descriptive statistics, and reliability evaluation. Structural equation modelling (SEM) was performed utilising AMOS version 28 to evaluate the proposed relationships among variables. Structural Equation Modelling (SEM) was selected for its capacity to examine intricate interactions, encompassing direct, indirect, and moderating effects, while considering measurement error (Byrne, 2022). The analysis employed a bifurcated methodology. Initially, validation of the measurement model was performed to ascertain construct validity, reliability, and model fit, which are essential for accurately depicting the study’s underlying constructs. Subsequently, structural model analysis was conducted to evaluate the proposed correlations among variables, yielding insights into both direct and indirect impacts. To strengthen the validity of the data, bootstrapping with 5,000 resamples was employed to evaluate the importance of mediation and moderation effects, in accordance with Hayes and Igartua (2021) guidelines. This method facilitated a thorough assessment of the proposed model, considering sampling variability.

### 3.6 Ethical considerations

This study complied with ethical standards to safeguard participants’ rights and confidentiality. Ethical approval was secured from the university’s ethics review board prior to data collection. Participants were provided with comprehensive information regarding the study, encompassing its objectives, methodologies, and voluntary participation. Electronic informed permission was secured prior to participants accessing the survey. Confidentiality was maintained by data anonymisation and secure storage on password-protected servers. No identifiable information was gathered, and participants were apprised of their choice to withdraw from the study at any moment without repercussions. The study conformed to the ethical principles specified in the Declaration of Helsinki and complied with data protection rules, including China’s Personal Information Protection Law (PIPL).

## 4 Results and discussion

This study’s results are organised to emphasise the reliability, validity, and robustness of the measurement model. This part offers a comprehensive study of demographic data, descriptive statistics, construct reliability and validity, assessment of discriminant validity, and model fit indices, accompanied by pertinent tables for clarity.

### 4.1 Demographic and descriptive analysis

The sample consisted of 320 respondents, achieving a response rate of 80%. Analysis of demographic data indicated variation in participant age, gender, educational attainment, and occupational responsibilities, so guaranteeing representativeness. Descriptive statistics for all constructs indicated that the data followed a normal distribution, with means between 3.8 and 4.2 and standard deviations under 1. These values demonstrate a balanced perception across constructs, affirming the lack of severe outliers and appropriateness for subsequent study.

### 4.2 Construct reliability and validity

All Cronbach’s alpha and composite reliability scores surpass the recommended level of 0.70, signifying robust internal consistency (Hair and Alamer, 2022). AVE values exceed the 0.50 threshold, so affirming convergent validity (Fornell and Larcker, 1981).

Table 1 provides a summary of Cronbach’s alpha, composite reliability (rho_c), and average variance extracted (AVE) for all constructs.

**Table 1:** Construct reliability and validity.

Construct	Cronbach’s alpha	Composite Reliability (rho_c)	AVE
Availability of family-friendly policies (AFP)	0.965	0.972	0.851
Emotional intelligence (EI)	0.949	0.959	0.797
Flexible work hours (FWH)	0.955	0.964	0.816
Stress levels (SL)	0.967	0.973	0.858
Support from school administration (SSA)	0.977	0.981	0.897
Teacher performance (TP)	0.972	0.977	0.876
Workload management (WM)	0.955	0.964	0.816

Source: Author’s research data.

### 4.3 Discriminant validity assessment

The Heterotrait-Monotrait Ratio (HTMT) was utilised to assess discriminant validity, with findings presented in Table 2. All HTMT values are below the 0.85 threshold, hence affirming discriminant validity (Henseler et al., 2015). This signifies that constructs are empirically independent and assess separate theoretical notions.

**Table 2:** Discriminant validity assessment and heterotrait-monotrait ratio of correlations (HTMT).

Construct pair	HTMT value
AFP–EI	0.846
AFP–SL	0.836
AFP–SSA	0.849
EI–FWH	0.763
SL–WM	0.735
SSA–TP	0.651

Source: Author’s research data.

### 4.4 Model fit

Model fit indicators were evaluated to determine the adequacy of the measurement model. The standardised root mean square residual (SRMR) was 0.025, significantly lower than the permitted limit of 0.08, signifying an exceptional fit (Hu and Bentler, 1999). The normed fit index (NFI) was 0.932, surpassing the minimum permissible threshold of 0.90. The elevated values of the model fit indices validate the robustness of the study framework. The results are encapsulated in Table 3.

**Table 3:** Model fit.

Fit Index	Value	Threshold
Standardised Root Mean Square Residual (SRMR)	0.025	< 0.08
Normed Fit Index (NFI)	0.932	> 0.90

Source: Author’s research data.

### 4.5 R-square and predictive relevance

The modified R-square values reflect the model’s predictive capability. The adjusted R-square for stress levels (SL) was 0.925, whereas teacher performance (TP) produced an adjusted R-square of 0.911. The values indicate that the independent factors account for a substantial percentage of the variance in the dependent variables, hence reinforcing the predictive significance of the framework. The findings validate the reliability, validity, and appropriateness of the measuring paradigm. The high internal consistency, acceptable discriminant validity, and superior model fit indices indicate the dataset’s sufficiency and the strength of the research methodology. These findings provide a robust basis for advancing with structural equation modelling (SEM) with SEM-PLS.

### 4.6 Structural equation model analysis

The direct relationship results present a nuanced view of the impact of work-life balance elements on teacher performance and stress levels. The presence of family-friendly policies (AFP) adversely affected stress levels (SL; *β* = −0.450, *p* < 0.001), suggesting that their absence intensifies stress. Nonetheless, AFP’s direct effect on TP (*β* = −0.098, *p* = 0.180) was not statistically significant. This study indicates that whereas AFP may mitigate stress, its direct effect on performance is mediated by other factors, consistent with COR theory, which posits that resource availability diminishes stress but does not directly improve performance.

**Figure 1:**
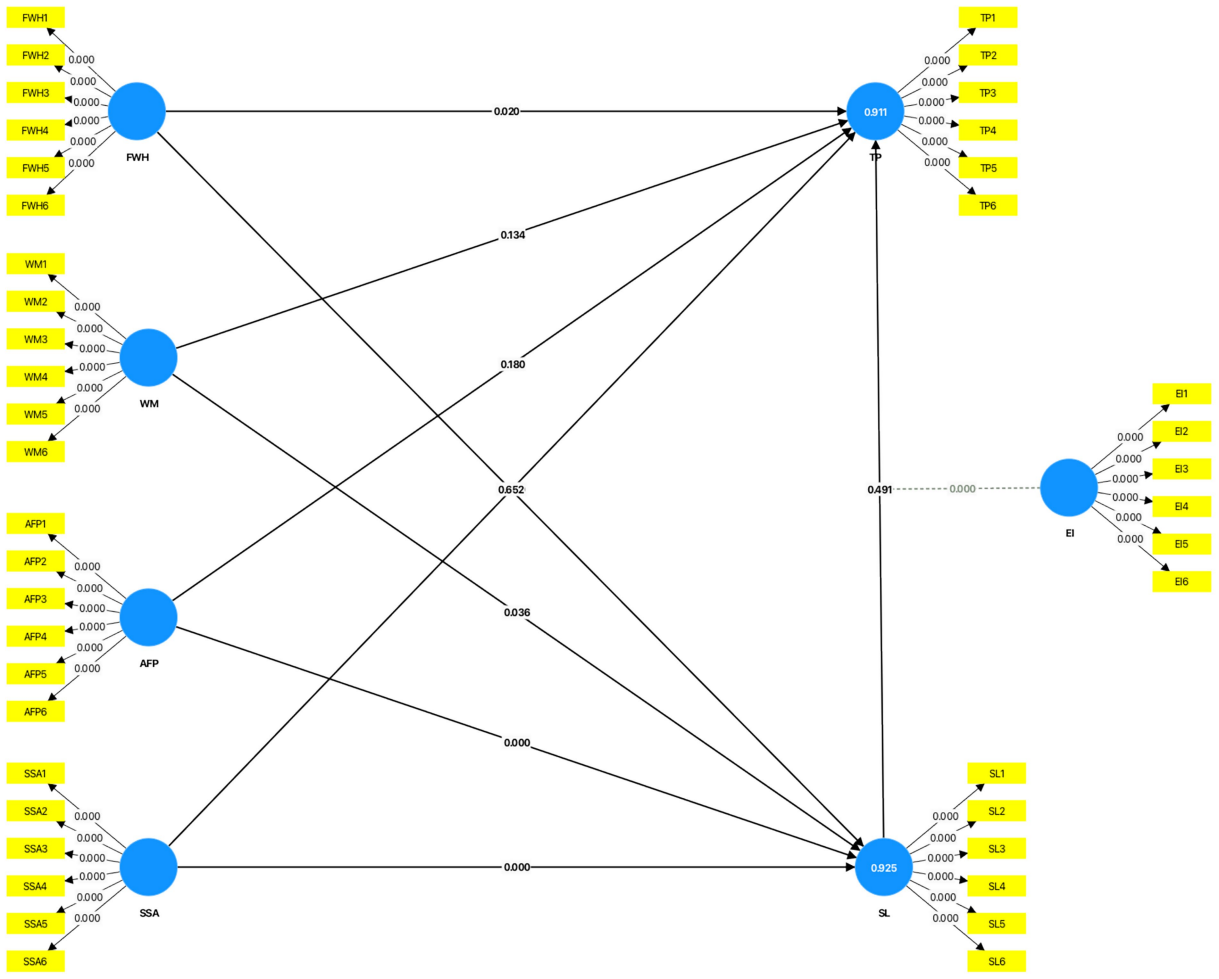
Graphical output—OL, *p*-value, R2 Adjusted. Source: Author’s research data.

Flexible work hours (FWH) significantly influenced teacher performance (TP; *β* = 0.234, *p* = 0.020), suggesting that flexibility facilitates effective resource allocation by educators, hence improving performance. Nonetheless, FWH did not exert a significant effect on SL (*β* = 0.061, *p* = 0.652), indicating that flexible hours alone may not resolve the underlying reasons of stress. Support from school administration (SSA) notably influenced both **stress levels** (SL; *β* = 0.327, *p* < 0.001) and teacher performance (TP; *β* = 0.204, *p* = 0.009), highlighting its dual function in alleviating stress and enhancing performance. Workload management (WM) significantly affected stress levels (SL; *β* = 0.155, *p* = 0.036), underscoring the necessity for efficient resource allocation to mitigate stress; however, its direct effect on task performance (TP; *β* = −0.118, *p* = 0.134) was not statistically significant. These findings corroborate other research highlighting the complex impact of work-life balance elements on teacher effectiveness (Li et al., 2025; Wang et al., 2024; Figure 1).

### 4.7 Indirect relationships: mediation analysis

The mediation study offered essential insights into the indirect mechanisms by which work-life balance issues affect teacher effectiveness. Stress levels (SL) fully mediated the relationship between AFP and TP (*β* = −0.170, *p* = 0.003), corroborating the COR theory’s claim that resource deficiency (e.g., absence of family-friendly policies) intensifies stress, therefore impairing performance. This study builds on previous studies by showing that AFP’s efficacy in enhancing TP depends on its capacity to alleviate stress (Stirling and Sangu, 2021).

Complementary partial mediation was identified in the association between SSA and TP via SL (*β* = 0.123, *p* = 0.010). SSA exerted both direct and indirect influence on TP, highlighting its dual function as a resource that mitigates stress while simultaneously offering support systems to improve performance. This discovery corresponds with the COR hypothesis, which asserts that resource acquisition not only alleviates resource depletion (stress) but also directly fosters favourable outcomes (Guo et al., 2024).

**Figure 2:**
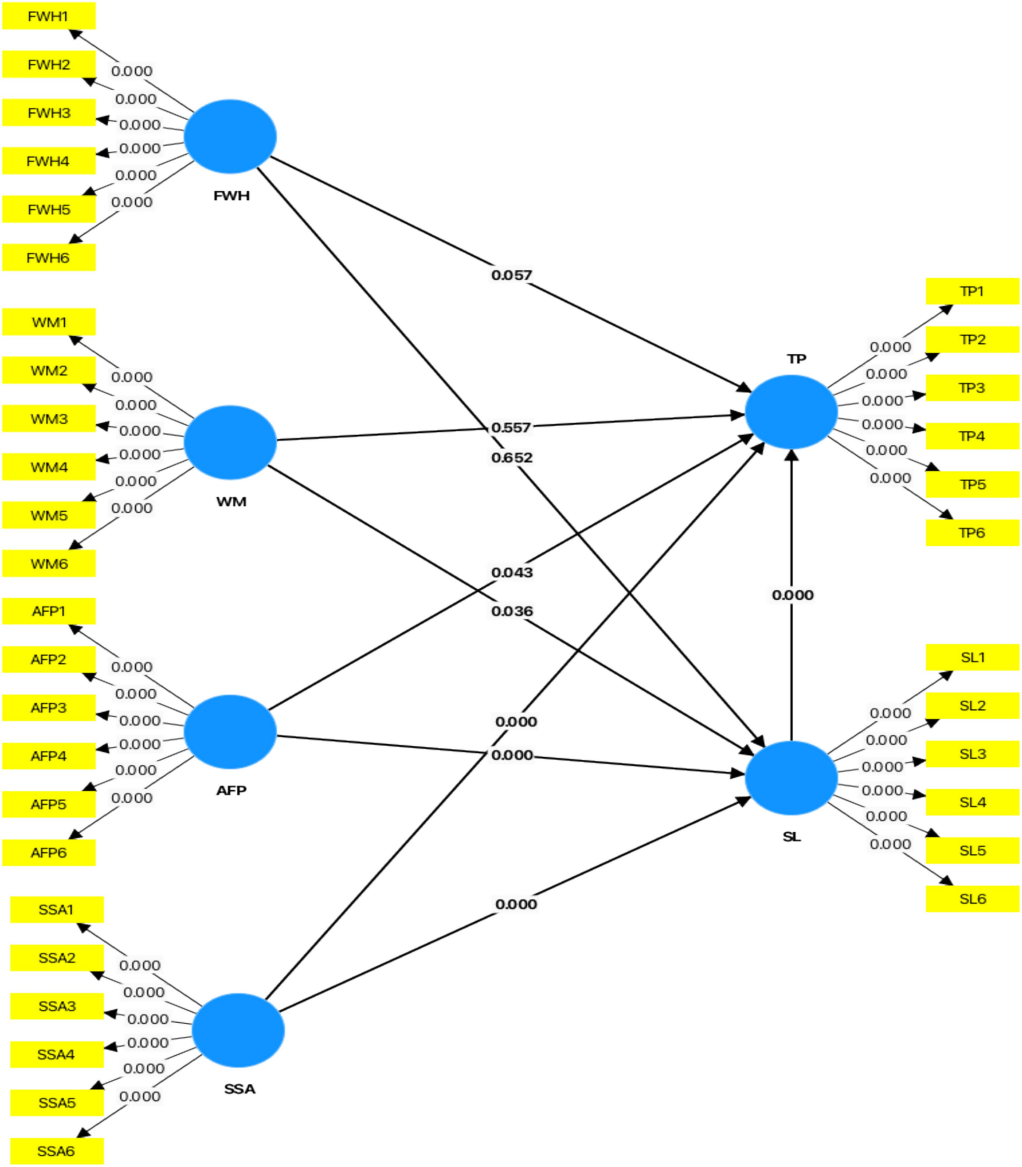
Graphical output (mediation analysis). Source: Author’s research data.

Unexpectedly, FWH and WM did not demonstrate substantial mediation via SL. For FWH, the indirect impact (*β* = 0.023, *p* = 0.628) was not significant, indicating that although flexibility directly improves performance, it does not meaningfully alleviate stress. Likewise, WM’s indirect effect (*β* = 0.059, *p* = 0.068) was marginally negligible; yet, its favourable influence on SL underscores its significance in resource management. The results somewhat contradict research highlighting the mediation function of stress in work-life balance scenarios (Rashmi and Kataria, 2021), suggesting that the impact of these elements may differ across various organisational environments and cultural contexts (Figure 2; Table 4).

**Table 4:** Mediation effect.

Total effect			Direct effect			Indirect effect							Hypothesis result
Coefficient	T value	*p* value	Coefficient	T value	*p* value	Hypothesis	Coefficient	SE	T value	*p* value	Percentile Bootstrap 95% CI		
											Lower	Upper	
0.239	2.371	0.018	0.234	2.326	0.020	H1: FWH→SL→TP	0.023	0.047	0.485	0.628	−0.063	0.121	No Mediation
−0.107	1.307	0.191	−0.118	1.500	0.134	H2: WM→SL→TP	0.059	0.032	1.823	0.068	0.012	0.146	No Mediation
−0.132	1.690	0.091	−0.098	1.341	0.180	H3: AFP→SL→TP	−0.170	0.056	3.021	0.003	−0.295	−0.072	Full Mediation
0.229	2.825	0.005	0.204	2.626	0.009	H4: SSA→SL→TP	0.123	0.048	2.574	0.010	0.048	0.248	Complementary Partial Mediation

Source: Author’s research data.

### 4.8 Moderation analysis: the role of emotional intelligence

**Figure 3:**
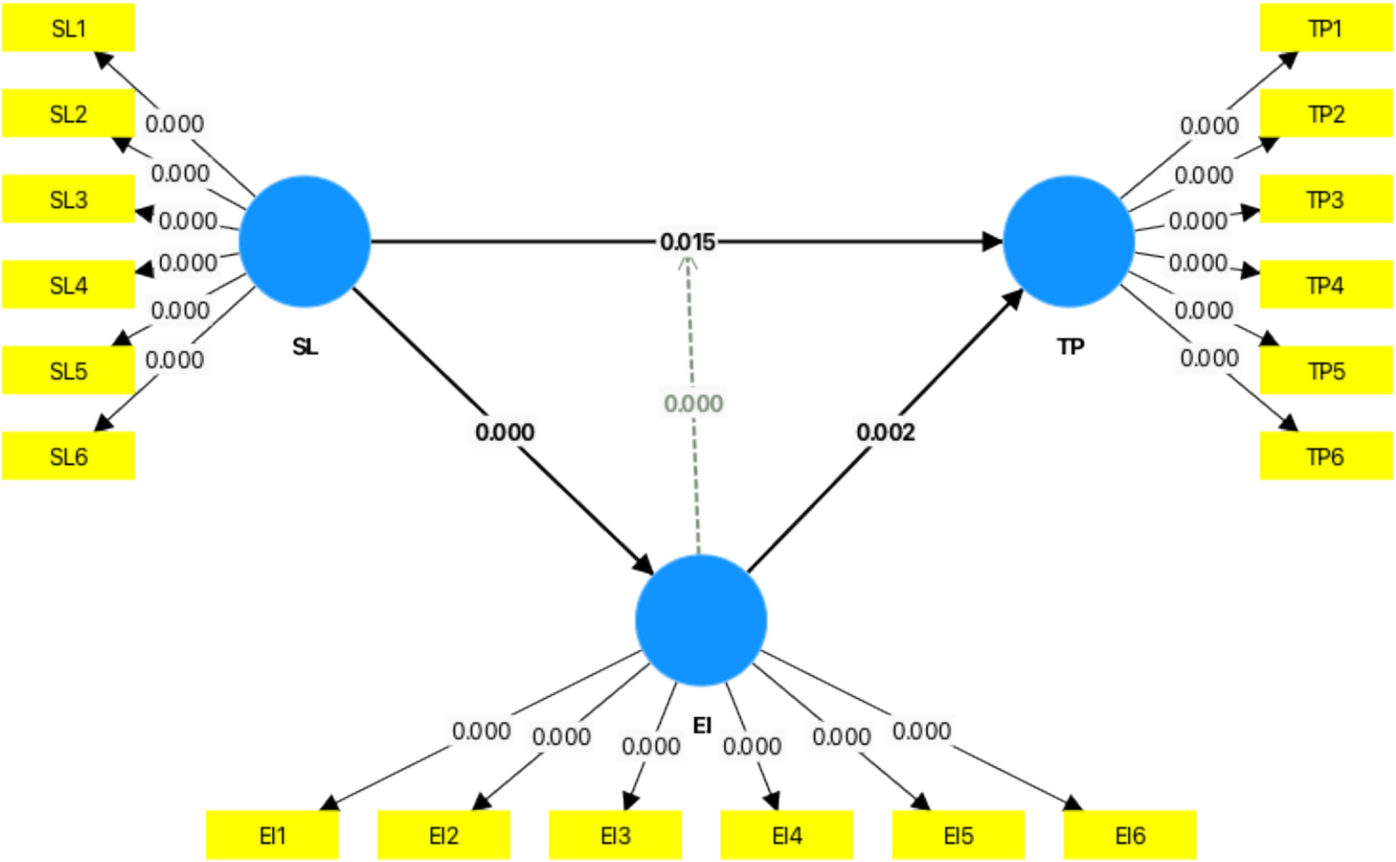
Graphical output (mediation analysis). Source: Author’s research data.

Emotional intelligence (EI) moderated the connection between stress levels (SL) and teacher performance (TP; *β* = −0.229, *p* < 0.001), indicating a buffering effect whereby elevated EI alleviates the adverse influence of stress on performance. This discovery highlights the significance of emotional intelligence as a personal asset that improves individuals’ capacity to manage stress efficiently, in accordance with the Conservation of Resources hypothesis (Hobfoll, 1989). Elevated emotional intelligence enables educators to manage their emotions, preserve resources, and sustain performance during stress, consistent with research by Khassawneh et al. (2022) (Figure 3; Table 5).

**Table 5:** Moderation effect.

Variables	Original sample (O)	Sample mean (M)	Standard deviation (STDEV)	T statistics (|O/STDEV|)	*p* values
EI x SL→TP	−0.229	−0.235	0.056	4.129	0.0

Source: Author’s research data.

## 5 Conclusion

This research investigates the mediating role of stress and the moderating influence of emotional intelligence within the framework of the Conservation of Resources (COR) theory, focussing on their impact on work-life balance and teacher effectiveness. The findings indicate that stress plays a significant mediating role in the relationship between resource loss and a reduced work-life balance, with emotional intelligence serving as a moderating factor that mitigates the effects of stress on performance outcomes. The results of this study broaden the applicability of COR theory within the educational domain, highlighting the influence of psychological and emotional resources on professional outcomes. The analysis contributed to the foundation for future research endeavours. Nonetheless, although the theoretical contributions are distinctly articulated, the practical implications of these findings are of considerable importance, providing avenues for the improvement of teacher well-being and the enhancement of institutional support frameworks.

## 6 Practical implications

The practical implications of this study are significant and warrant careful consideration. The findings provide valuable insights that can inform future research and practice in the relevant field. Furthermore, the application of these results may enhance understanding and contribute to the development of effective strategies. The findings of this study provide practical implications for educational institutions and policymakers. Initially, it is imperative to prioritise stress-reduction initiatives, including workload management, counselling services, and peer support programmes, to effectively mitigate resource depletion among educators. Furthermore, the implementation of emotional intelligence training programmes has the potential to provide educators with essential coping strategies, thereby enhancing their ability to manage stress and optimise classroom effectiveness. Third, the promotion of a supportive organisational culture—facilitated by policies such as flexible scheduling and recognition systems—has the potential to improve work-life balance and enhance job satisfaction. Ultimately, the implementation of systematic well-being assessments enables educational institutions to identify educators who may be at risk and to design targeted interventions in a proactive manner. The implementation of these measures enhances the resilience of individual educators while simultaneously fostering greater institutional success through the mitigation of burnout and turnover rates. Considering the pivotal function of educators in influencing future generations, the allocation of resources towards such interventions emerges as a strategic and ethical necessity.

## 7 Limitations

The present study acknowledges certain limitations that may impact the interpretation of the findings. These constraints warrant careful consideration in future research endeavours.

Notwithstanding its contributions, this study presents several limitations that merit careful consideration. Initially, the geographical constraints of the sample may impose limitations on the generalisability of the findings to alternative regions or cultural contexts. Subsequent investigations ought to encompass a variety of populations to ascertain the model’s generalisability. Furthermore, the dependence on self-reported data presents a risk of response bias; the incorporation of objective measures, such as administrative data or observational assessments, may enhance the reliability of the findings. Third, the cross-sectional design limits the ability to draw causal inferences; therefore, longitudinal studies are essential to investigate the dynamic interplay between stress and emotional intelligence over time. In conclusion, although the emphasis on educators yields valuable insights pertinent to the sector, extending this inquiry to encompass additional high-stress professions, such as those in healthcare or corporate environments, may reveal more generalised trends in the dynamics of resource conservation. Future research endeavours aimed at addressing these limitations will contribute to the refinement of both the theoretical and practical applications of COR theory.

## Data Availability

The original contributions presented in the study are included in the article/supplementary material, further inquiries can be directed to the corresponding author.
